# Does the Modification of the Apical Geometry of a Dental Implant Affect Its Primary Stability? A Comparative Ex Vivo Study

**DOI:** 10.3390/ma14071728

**Published:** 2021-04-01

**Authors:** Henning Staedt, Diana Heimes, Karl M. Lehmann, Peter Ottl, Monika Bjelopavlovic, Wilfried Wagner, Bilal Al-Nawas, Peer W. Kämmerer

**Affiliations:** 1 Private Practice, Blumenstraße 43, 73728 Esslingen am Neckar, Germany; henning@staedt.com; 2 Department of Prosthodontics and Materials Science, University Medical Center Rostock, Strempelstraße 13, 18057 Rostock, Germany; peter.ottl@med.uni-rostock.de; 3 Department of Oral- and Maxillofacial Surgery, University Medical Center Mainz, Augustusplatz 2, 55131 Mainz, Germany; Wilfried.wagner@unimedizin-mainz.de (W.W.); bilal.al-nawas@unimedizin-mainz.de (B.A.-N.); peer.kaemmerer@unimedizin-mainz.de (P.W.K.); 4 Department of Prosthetic Dentistry, University Medical Center Mainz, Augustusplatz 2, 55131 Mainz, Germany; karl.lehmann@unimedizin-mainz.de (K.M.L.); monika.bjelopavlovic@unimedizin-mainz.de (M.B.); 5 Department of Life, Light & Matter, University of Rostock, Albert-Einstein-Straße 25, 18059 Rostock, Germany

**Keywords:** dental implant primary stability, camlog screw-line promote^®^ plus, apical screw thread, bone density, J-line, K-line

## Abstract

(1) Background: Primary stability—one fundamental criterion for the success of dental implants—is influenced by implant geometry even if the effect of apical shape modifications on implant primary stability has not yet been examined. Therefore, the aim of the ex vivo study was to compare primary stability of implants differing in apically located screw threads (J-line) or a flat tip (K-line) only. (2) Methods: 28 implants of each group of the same diameter (4.3 mm) were randomly inserted into porcine bone blocks. The first group (9, 11 and 13 mm) was inserted into “hard”, the second (11 mm) into “soft” bone, here using a normal and an undersized drilling protocol. Insertion torque (Ncm), Periotest^®^ value, resonance frequency (implant stability coefficient, ISQ) and push-out force (N) were measured. (3) Results: In “hard” bone, primary stability increased with increasing length in both groups but it was significantly higher in J-line (*p* < 0.03). An undersized preparation of the implant bed in “soft” bone resulted in a significant increase in primary stability in both groups. Here, J-line also showed a significantly increased primary stability when compared to equally prepared K-line (insertion torque: 37 Ncm vs. 26 Ncm; Periotest^®^: −6.5 vs. −4.3; push-out force: 365 N vs. 329 N; *p* < 0.05 each). (4) Conclusions: Primary stability is significantly higher with increasing implant length and apically located screw threads as well as with undersized drilling protocols. When preparing the implant site and subsequently selecting the implant system, modifying factors such as implant geometry (also at the tip) should be taken into account.

## 1. Introduction

The insertion and restoration of dental endosseous implants is an integral part of treatment for partial and total edentulism. The affected patients benefit from increased masticatory and esthetic performance while maintaining osseous and soft tissue sites [1,2]. Primary stability—an essential determinant of subsequent osseointegration and thus a fundamental criterion for the success of dental implants [3]—is defined as the absence of mobility in the bone bed after placement. It is, rather, related more to mechanical immobility (i.e., friction between bone and implant during insertion) than to histological criteria and can thus be clearly distinguished from secondary stability, which is generated by osseous regeneration and remodeling during the healing process. While primary stability is influenced by the surgical technique, the quality/quantity of the bone and the geometry or design of the implant, secondary stability is determined by bone regeneration and remodeling at the peri-implant bone site [3,4,5].

In the posterior maxilla in particular, a thin cortical bone is often present together with a trabecular core of lower density (type IV according to Lekholm and Zarb) [6]. Here, sufficient primary stability might be difficult to achieve. Therefore, modifications of the drilling technique or the macrodesign of the implant are recommended [7,8,9]. In regard to drilling techniques, undersized preparation creates an implant bed with a diameter that is significantly smaller than the implant diameter, resulting in an increase in primary stability [8,10,11]. However, Campos et al. were able to show in an animal model that although the undersized preparation resulted in a high insertion torque, new bone formation was highest in the group with a normal drilling protocol. Accordingly, the authors concluded that high values for insertion torque do not necessarily lead to the most favorable biological response [12]. The implant design is one of the key factors affecting primary stability and stress distribution on the peri-implant hard tissue. Recently, numerous in silico, in vitro, ex vivo and in vivo studies have investigated the effect of the implant’s geometry on the surrounding peri-implant tissue. Frequently, parameters such as the diameter, length, bevels or screw threads were investigated with some general trends being observed: diameter and length had a strong effect on the mechanical peri-implant environment. Increasing length, for example, produced a minimal stress in cancellous bone whereas an increased diameter of the implant resulted in minimum stress in the cortical bone [13]. Implants with deeper screw threads, smaller pitch and helix angle have been shown to increase primary stability by achieving higher bone-to-implant contact while reducing osseocompression [14,15,16]. In contrast, Makary et al. provided evidence that the use of implants with deep screw threads could be beneficial in implant rehabilitations in soft bone (D3 and D4) only [17]. Altogether, an ideal implant design should allow a balance between compressive and tensile forces while minimizing the generation of shear forces [18,19,20].

Currently, implant insertion torque measurements and the use of resonance frequency analysis (RFA) are the most widely accepted biomechanical parameters for measuring primary stability [7,10,21]. However, the two indicators differ in terms of their approach: while insertion torque measures the resistance arising when the implant is inserted in the apical direction, the RFA measurement records the natural oscillation frequency of the implant in the bone, which depends on the stiffness of the implant-bone connection. The Periotest^®^ device was developed and described as an electromechanical instrument for measuring implant stability [22,23,24]. In brief, it uses percussion of the test object by an electrically driven plunger whose pressure-sensitive head measures the contact time with the test object. Furthermore, via measurement of push-out force—a destructive, maximally invasive parameter—the friction force of the implants can be determined indirectly [25].

The modification of the apical implant design regarding a reduction of the apical screw threads between 2002 and 2009 by Camlog (Camlog Biotechnologies AG, Basel, Switzerland) raised the question of the effect of this variation on the implants’ primary stability. Considering the limited evidence on this topic, the aim of this study was to determine whether differences in the apical geometry of dental implants, particularly the extension of the cutting thread to the implant apex, lead to a change in primary stability. As a secondary parameter, the influence of the two apical geometries was to be measured when using two different drilling protocols to prepare the implant bed in softer bone. The null hypothesis was that the extension of apical screw threads would not result in increased primary stability.

## 2. Materials and Methods

### 2.1. Implant Design

**Figure 1:**
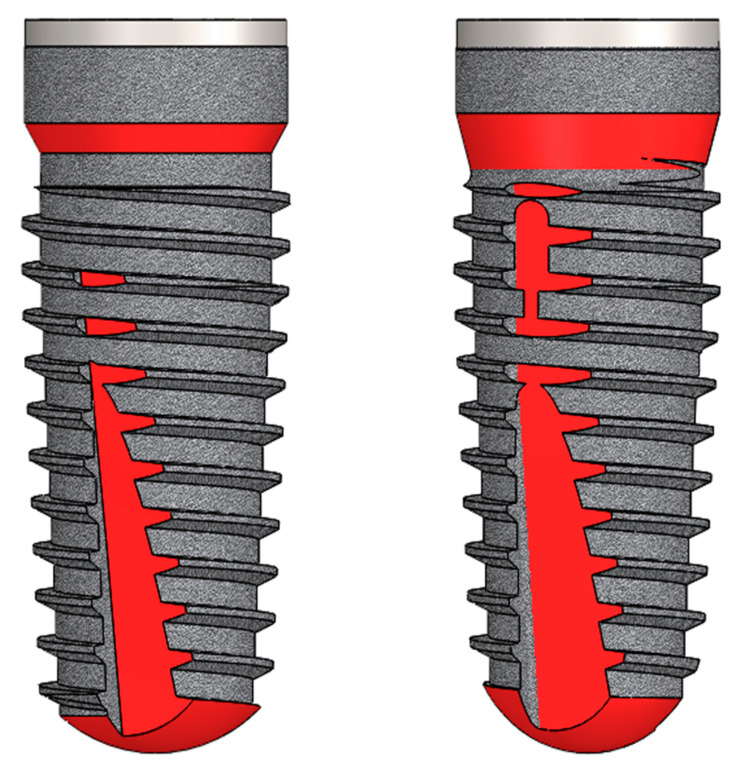
Schematic illustrations of the two implant systems (Camlog Screw-Line, Promote^®^ plus) showing the J-line (**left**) and the K-line (**right**). Changes in macrogeometry are marked in red (with kind approval: Camlog Biotechnologies AG, Basel, Switzerland).

The Swiss-German Camlog Group (Camlog Biotechnologies AG, Basel, Switzerland) introduced the screw-line implant (J-line) in 2002 and expanded it to the K-line in 2009. In general, screw-line implants are made out of grade 4 titanium and have a slightly tapered outer geometry (3°–9°), a self-tapping thread and a machined implant neck portion. The surface of the implants (Promote^®^ (Camlog Biotechnologies AG, Basel, Switzerland)) is blasted and acid-etched. From the J- to the K-line, there were three main adjustments in the external macrodesign:

1. while in the J-line the cutting threads extended to the end of the implant, in the K-line they were shortened, resulting in an apical rounding,
2. the cutting part of the implant was elongated cranially from J- to K-line and
3. in the K-line, a shorter cylindrical portion was designed crestally with a beveling of the former taper there (Figure 1, changes highlighted in red).

### 2.2. Specimens

As already published by our group [5,26,27], 15 mandibular bones of fresh porcine cadavers were purchased from a local butcher. According to the butcher, the animals showed no signs of pathological bone conditions. The mandibular bones were cut in half using a water-cooled precision saw (EXAKT^®^ Sawing-Grinding System; EXAKT, Norderstedt, Germany). Subsequently, the bone quality was radiologically assessed on each specimen by means of cone beam computed tomography (CBCT).

Subsequently, using the CBCT data, blocks (30 mm × 70 mm × 20 mm) were taken from the mandibular bone sections, which had to have CBCT ratios of white and black pixels of 0.33 (±0.1) (mixed cortical-spongy bone (“hard” bone)) or a ratio of 0.15 (±0.1) (cancellous bone (“soft” bone)) [25,26]. In order to avoid inaccuracies, another method of measuring bone quality was subsequently performed using ultrasound transmission velocity, with “hard” bone having a value of 1472 m/s (±60) and “soft” bone having a value of 1267 m/s (±130) [25]. After removal of the surrounding soft tissue, the surfaces of the bone specimens were thoroughly cleaned by water rinsing and macroscopically checked for irregularities. In 20 bone blocks (“hard” *n* = 10, “soft” *n* = 10) no irregularities were present in combination with a minimum thickness of 20 mm. These were finally included in the experiment and immediately randomized for further investigations [5].

### 2.3. Surgery

**Figure 2:**
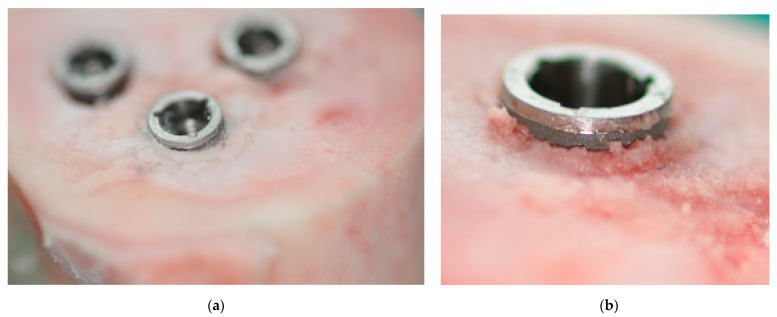
(**a**) Supracrestal insertion of three implants into a “soft” bone block. (**b**) Close-up of supracrestal insertion into a “soft” bone block.

Fifty-six implants (Screw-Line, Promote^®^ plus) with a diameter of 4.3 mm and lengths of 9, 11 and 13 mm were studied, with a total of 28 implants from the J-line (4.3 mm × 9 mm *n* = 4; 4.3 mm × 11 mm *n* = 20; 4.3 mm × 13 mm *n* = 4) and 28 implants from the K-line (4.3 mm × 9 mm *n* = 4; 4.3 mm × 11 mm *n* = 20; 4.3 mm × 13 mm *n* = 4). Three to four implants were randomly placed supracrestally in each bone block as it was intended to rule out the effect of the cranial changes in implant geometry (Figure 2).

**Figure 3:**
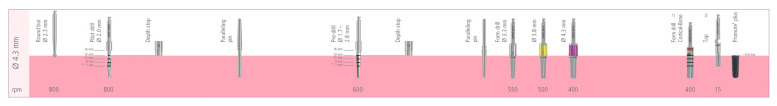
Drilling protocol for Camlog Screw-Line, Promote^®^ plus (with kind approval: Camlog Biotechnologies AG, Basel, Switzerland).

The experiments were divided into two parts:

Part A: J-line and K-line implants of the same diameter (4.3 mm) and different lengths (9, 11, and 13 mm) were inserted into “hard” bone blocks (*n* = 4 per group) according to the manufacturer’s instructions. In detail, after marking the desired implant position with the rose drill (diameter 2.3 mm), deep drilling with the pilot drill (diameter 2 mm), predrilling with the pilot drill (diameter 1.7–2.8 mm) and final form drilling (3.3, 3.8 and 4.3 mm) were performed. Now the implant was inserted with the machine. It should be noted that in all implant insertions the insertion depth was 0.4 mm less than specified by the manufacturer, so that the implants could be placed supracrestally as explained above (Figure 3).

Part B: J-line and K-line implants of the same diameter (4.3 mm) and length (11 mm) were placed using two insertion techniques (normal drilling (see above), undersized drilling (final drilling with the 3.8 mm diameter form drill)) in “soft” bone. Each experiment was repeated with *n* = 4 per group. Again, the insertion was supracrestal.

### 2.4. Measurements

After mechanical insertion of the implants (Elcomed, type SA-310, W&H Dentalwerk Bürmoos GmbH, Bürmoos, Austria), the maximum insertion torque was recorded with a dynamometric ratchet (Mecmesin^®^, Schwenningen, Germany [28]) (values Ncm). After insertion of the respective implant, the following values were recorded:

- Electromechanical measurement of implant stability using the Periotest^®^ device over 4 s with *n* = 3 measurements per implant [29,30]. For this purpose, the abutments of the implants were inserted according to the manufacturer’s instructions. The Periotest^®^ value scale ranges from −8 to +50, with smaller Periotest^®^ values representing greater stability of the measured object. Mean values were used for the statistical evaluations.
- Bone resonance frequency measurement (RFA) using the Osstell^®^ resonance frequency analyzer (Integration Diagnostics, Gothenburg, Sweden). For this purpose, the abutments were carefully removed, the corresponding transducers were mounted on the implants and the Ossell^®^ device was positioned at a distance of 3 mm from the transducer. The recorded frequencies (*n* = 3 in vertical and in horizontal orientation per implant) were automatically converted into ISQ (implant stability coefficient) values in the range of 0–100 (minimum to maximum stability) [27,31]. Mean values were used for the statistical evaluations.
- After performing the aforementioned measurements, all implants were pushed out using a Zwick UPM (Universalprüfmaschine) materials testing machine (Zwick, Atlanta, GA, USA). For this purpose, axial compression forces (continuously 0.5 mm/min) were applied to the cranial end of each implant and the shear force to detach the implant from the bone was recorded in Newtons (N) [22]. Mean values were used for the statistical evaluations.

### 2.5. Statistics

A sample size of *n* = 4 was chosen that is higher that sample sizes reported in another similar study on primary stability testing [25]. Even so, due to the low number of implants per group, reports of statistical significance level have to be considered to be descriptive only. The test parameters of insertion torque (Ncm), Periotest^®^ and ISQ values, and push-out force (N) were compared between the groups in parts A and B, respectively. Raw data sets were stored in Excel^®^ spreadsheets (Microsoft Corporation, Redmond, WA, USA) and then transferred to SPSS Statistics ^®^ (version 24, macOS X, SPSS Inc., IBM Corporation, Armonk, NY, USA). Data were expressed primarily as means and standard deviations. Normal distribution of data was checked using a non-parametric Kolmogorov–Smirnov test. Results were analyzed for statistical significance using an analysis of variance (ANOVA), unpaired non-parametric Mann–Whitney U tests, Wilcoxon Whitney tests, and Student’s *t*-tests. Descriptive statistical significance level was set at *p* ≤ 0.05 and boxplots were used for illustrative purposes.

## 3. Results

### 3.1. Part A

The original data for the measurements of part A can be found in Supplementary Materials Table S1.

#### 3.1.1. Insertion Torque

**Figure 4:**
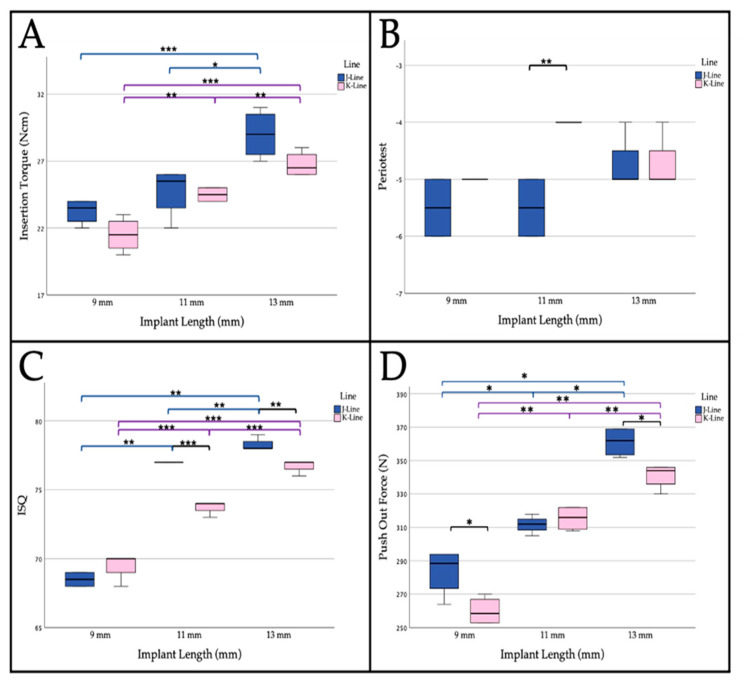
Boxplots illustrating (**A**) insertion torque, (**B**) Periotest^®^ measurement, (**C**) resonance frequency analysis and (**D**) push-out forces of the J- and K-lines at different implant lengths in “hard” bone. Within-group significance in assigned colors (J-line blue, K-line pink) and between-group significance is assigned in black. Shown are means ± SD, * p < 0.05, ** p > 0.01, *** p < 0.001.

In total, the measurements in “hard” bone showed a mean value of 25.7 Ncm (standard deviation (SD): 2.9 Ncm) for J-line and a mean value of 24 Ncm (SD: 2.4 Ncm) for K-line without statistically significant difference (*p* = 0.21). Including the implant lengths, an insertion torque of 23.3 Ncm (SD: 0.96 Ncm) was found for the implants with 9 mm length for the J-line compared to the K-line with 21.5 Ncm (SD: 1.3 Ncm; *p* = 0.072). There were no statistically significant differences between the two lines with lengths of 11 mm (J-line mean 24.75 Ncm (SD: 1.9 Ncm), K-line mean 24.5 Ncm (SD: 0.6 Ncm; *p* = 0.809)). In implants of 13 mm length, the differences were also not statistically significant (J-line mean 29 Ncm (SD: 1.8 Ncm), K-line mean 26.8 Ncm (SD: 0.96 Ncm; *p* = 0.072)). However, the mean values of the insertion torque of J-line were always slightly higher when compared to those of K-line. Furthermore, there was a steady increase in insertion torque with increasing implant length, regardless of the implant line. In the J-line, the increase from 9 mm to 11 mm was not (*p* = 0.207), but the difference between 9 and 13 mm (*p* = 0.001) and the difference between 11 and 13 mm (*p* = 0.018) was significantly different. In K-line, statistically significant differences were present between 9 and 11 mm (*p* = 0.005), 9 and 13 mm (*p* = 0.001), and between 11 and 13 mm (*p* = 0.007) (Figure 4A).

#### 3.1.2. Periotest^®^ Measurement

In “hard” bone—regardless of the implant length—significant lower values were measured for J-line (J-line mean −5.3 (SD: 0.62), K-line mean −4,6 (SD: 0.5); *p* = 0.009). However, no significant differences were present at 9 mm (J: −5.5 (SD: 0.58); K: −5 (SD: 0.0); *p* = 0.134) and 13 mm (J: −4.75 (SD: 0.5); K: −4.75 (SD: 0.5); *p* = 1) lengths. In contrast, the differences between J- and K-line were significantly different at implant lengths of 11 mm (J: −5.5 (SD: 0.56); K: −4 (SD: 0.0); *p* = 0.002) (Figure 4B).

#### 3.1.3. Resonance Frequency Analysis

In total, no significant differences were found in ISQ values between J- and K-lines (J: 74.6 (SD: 5.4); K: 73.3 (SD: 3.2); *p* = 0.443). Similar to the insertion torque, ISQ values generally showed increasing values with increasing implant length. Comparing the J-line implants with each other, there was a mean ISQ value of 68.5 (SD: 0.58) for the 9 mm implants, 77 (SD: 0) for the 11 mm implants, and 78.3 (SD: 0.5) for the 13 mm implants. Between all groups, the differences were statistically significant (*p* < 0.003 in each case). Statistically significant differences were also present within the K-line between the different lengths (9 mm: 69.5 (SD: 1); 11 mm: 73.75 (SD: 0.5); 13 mm: 76.75 (SD: 0.5); each *p* < 0.001; Figure 4C). Direct comparison between J- and K-line implants at 9 mm length showed no significant difference (J: 68.5 (SD: 0.6); K: 69.5 (SD: 1); *p* = 0.134). In contrast, J-line implants at 11 mm length had significantly increased ISQ values when compared to K-line (J: 77 (SD: 0); K: 73.8 (SD: 0.5); *p* < 0.001). The same tendency was observed for implants with the length of 13 mm (J: 78.25 (SD:0.5); K: 76.8 (SD: 0.5); *p* = 0.005).

#### 3.1.4. Push-Out Force

For push-out force in general, no significant differences were found between the two implant lines (J: 312.2 N (SD: 26 N); K: 312.3 N (SD: 43.9 N); *p* = 0.893). A steady increase in primary stability with increasing implant length was seen for all implants. This was statistically significant both within the J- and within the K-line between implants of different lengths. Specifically, a mean of 283.8 N (SD: 14.2 N) was measured for the J-line at 9 mm, 311.8 N (SD: 5.3 N) at 11 mm, and 361.3 N (SD: 9 N) at 13 mm (between groups *p* < 0.02). For the K-line, the mean values were 260 N (SD: 8.4 N) at 9 mm length, 315.5 N (SD: 7.5 N) at 11 mm, and 341 N (SD: 7.6 N; between groups *p* < 0.005; Figure 4D). In addition, significantly higher push-out forces were seen for 9 mm (J: 283.8 N (SD: 14.2 N); K: 260 N (SD: 8.4 N); *p* = 0.028) and 13 mm (J: 361.3 N (SD: 9 N); K: 341 N (SD: 7.6 N); *p* = 0.014) implants in the J- compared to the K-line. No significant difference was seen in 11 mm implants (J: 311.8 N (SD: 5.3 N); K: 315.5 N (SD: 7.6 N); *p* = 0.448; Figure 4D).

### 3.2. Part B

The original data for the measurements of part B can be found in Supplementary Materials Table S2.

#### 3.2.1. Insertion Torque

**Figure 5:**
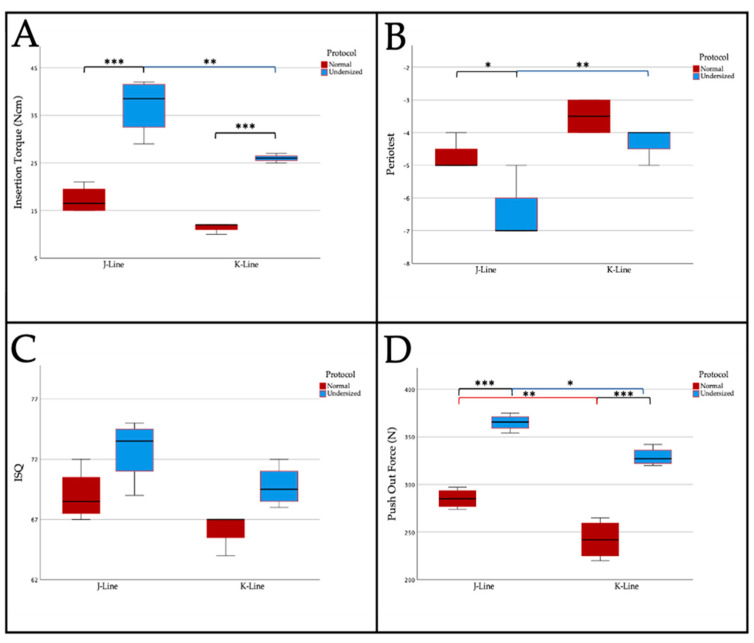
Boxplots illustrating (**A**) insertion torque, (**B**) Periotest^®^ measurement, (**C**) resonance frequency analysis and (**D**) the push-out forces of the J- and K-line for different drilling protocols in “soft” bone. Within-group significance in assigned colors (J-line blue, K-line pink) and between-group significance is assigned in black. Shown are means ± SD, * *p* < 0.05, ** *p* > 0.01, *** *p* < 0.001.

In the J-line group, the standard drilling protocol in “soft” bone showed a mean insertion torque of 17.3 Ncm (SD: 2.9 Ncm) while in the undersized group there was a significantly higher insertion torque (37 Ncm (SD: 5.9 Ncm); *p* < 0.001). A similar outcome was observed in the K-line group (Normal (N): 11.5 Ncm (SD: 1 Ncm); Undersized (UD): 26 Ncm (SD: 0.8 Ncm); *p* < 0.001). Comparing the two lines, J-line showed no significant difference in normal preparation compared to normally prepared K-line implants (*p* = 0.195). In contrast, J-line implants showed significantly higher insertion torques after undersized preparation when compared to undersized prepared K-line implants (*p* = 0.004; Figure 5A).

#### 3.2.2. Periotest^®^ Measurement

The measurement of Periotest^®^ values showed significant differences (*p* = 0.02) in J-line between normal (mean −4.8 (SD: 0.5)) and undersized (mean −6.5 (SD: 1) prepared implant beds. In contrast, such a difference could not be verified for K-line (N: −3.5 (SD: 0.58); UD: −4.3 (SD: 0.5)). When comparing the normally prepared J-line implants with their K-line counterparts, there were also no significant differences (*p* = 0.137). In contrast, undersized preparation resulted in significantly lower Periotest^®^ values for the J-line when compared to the K-line (*p* = 0.003; Figure 5B)

#### 3.2.3. Resonance Frequency Analysis

For ISQ scores, no statistically significant difference was seen for J-line (N: 69 (SD: 2.2); UD: 72.8 (SD: 2.6); *p* = 0.142) and K-line implants (N: 66.3 (SD: 1.5); UD: 69.8 (SD: 1.7); *p* = 0.194) in relation to drilling protocol (Figure 5C).

#### 3.2.4. Push-Out Force

J-line implants showed significantly lower push-out values after using the normal drilling protocol when compared to the undersized drilling protocol (N: 285.3 N (SD: 10.2 N); UD: 365 N (SD: 8.7 N); *p* < 0.001). A correspondingly significant change was also observed in the K-line (N: 242.3 N (SD: 20.8 N); UD: 329 N (SD: 9.6 N); *p* < 0.001). There were equally significant differences between the normally prepared J-line and K-line implants as well as between the two undersized prepared groups (*p* = 0.004 & *p* = 0.014; Figure 5D).

## 4. Discussion

The present study is based on the fact that there is a relationship between implant shape and shear strength during implant placement that modulates bone compression. In brief, the study was conducted in order to investigate if changes in the apical geometry of an implant system can influence the primary stability of implants in bone of lower and higher density. To obtain a high degree of diagnostic certainty, four different methods of measuring primary stability were used for this purpose. As a secondary outcome parameter, possible differences in primary stability of apically modified implants were investigated when using two different drilling protocols in soft bone. To the best of the authors’ knowledge, these are the first experiments that specifically addressed apical modifications of dental implants in combination with an evaluation of primary stability. The results of this work confirmed the significant influence of the apically located macrogeometry of a dental implant on primary stability. Therefore, the null hypothesis had to be rejected.

Due to its structural similarity to human bone, porcine bone was used in the present study, which is also used in pre- and postgraduate training courses [32]. Bone quality describes the proportion of cortical cancellous bone as well as it density. Many studies have shown that bone quality has a significant impact on implant survival. According to Lekholm and Zarb, different types can be distinguished. In type I-bone, the entire bone is composed of very thick cortical bone. Type II consists of a thick layer of cortical bone surrounding a core of dense trabecular bone. Type III is composed out of a thin layer of cortical bone surrounding a core of trabecular bone of good strength and Type IV is defined to have a very thin layer of cortical bone with low density trabecular bone of poor strength. The bone quality at the site of the planned implant insertion determines the primary stability of an implant and is a procedure-dependent local factor for the survival and success of the inserted implant [22,30]. Type IV-bone, in particular, is associated with a high rate of implant loss [33]. Bone density can be measured objectively using several techniques, which can be divided into invasive and non-invasive. Invasive approaches include bone sampling during implant osteotomy preparation followed by histological examination. Also included are radiological three-dimensional images such as CT, CBCT or micro-CT, which allow reconstruction of the bone with evaluation of various structural parameters such as bone density, porosity, and trabecular thickness [25]. Ultrasound transmission velocity represents a non-invasive, objective technique for determining bone quality, which has been verified in previous studies [25,26,34]. In order to perform a uniform characterization of the bone used in this study, a combination of invasive, 3D radiological and non-invasive diagnostic tools (ultrasound transmission velocity) was used.

Even if the primary stability of dental implants is controversially discussed as a decisive parameter for successful masticatory functional rehabilitation in the context of two-stage loading concepts and oversized drilling protocols are also employed, the development of alternative loading concepts (i.e., transgingival healing, immediate loading) showed the need as well as the importance of primary stability [27]. It has been repeatedly found in the literature that the final osteotomy position for the preparation of the implant bed should be determined by adapted drilling in order to achieve an individual and variable degree of bone compression [35]. In this context, several modifications of surgical techniques have been proposed, which include undersized drilling protocols, bone condensation, and/or bicortical fixation [11]. Undersized preparation of the drill cavity appears to be one of the most efficient and easiest ways to increase primary stability [36].

Due to the direct implant–bone contact and their ankylotic fixation, dental implants show a different biomechanical behavior when compared to natural teeth. Finite element analyses could give evidence for the development of stress under loading conditions and were able to identify high stress peaks in the cortical region while the cancellous bone is relieved. The cortical bone has an E-modul 10 times higher than that of the cancellous bone and can thus offer high resistance to the horizontal and vertical loading of the implant. For this reason, high stress peaks occur around the implant neck, which are suspected of inducing crestal bone resorption. By increasing the stiffness of the implant, however, these stress peaks can be reduced. An increase in implant stiffness can be achieved mathematically by increasing the implant diameter. The design of the implant is another way to influence the stress distribution. The largest possible surface with a high degree of bone contact to minimize the specific surface pressure can be achieved, for example, by using threads or microstructuring the implant’s surface [33,37]. According to the mathematical calculations, we were able to show that an increasing implant length correlates with an increase in primary stability. Similarly, Arosio et al. concluded in their in vitro study that the increase in implant diameter or length results in greater primary stability in case of higher bone densities [38]. Mesa et al. performed a retrospective analysis of the primary stability of 1084 implants in 316 patients and found a significant association between Periotest^®^ values and implant lengths [39], whereas Ostman et al. calculated a significant association between ISQ values and implant length and diameter [40]. Silva et al. compared very short and short implants ex vivo and could conclude—also analogous to the present study—that longer implants (and a beveled design) lead to better primary stability [41]. In contrast, other groups in prospective and retrospective studies failed to identify any corresponding correlations [42,43]. Some of these discrepancies can be explained by different types of bias. For example, different implant designs were used, the manufacturer’s insertion protocol or a customized one was applied, and the implants were placed in different jaw areas with different bone qualities. In the Periotest^®^ measurements, different variables such as changes in the implant diameter, the vertical measuring point on the implant abutment and the attachment angle as well as the horizontal distance between the measuring head and the implant abutment can also have an influence on the measurements. [22]. Also in the present study, the influence of such variables cannot be excluded although the study was conducted by one investigator only, using pre-defined bone samples and one implant type with slight modifications. Thus, the variability was kept as low as possible.

One possibility for changing the implant geometry is the use of apically located, (self-tapping) threads, whereby self-tapping implants showed partially better but also partially worse implant stability with similar clinical success when compared to non-self-tapping implants [22,44]. The apical geometry of an implant has an influence on the retention force in the bone and thus on the primary stability, as demonstrated in the present study. Even though this part of the geometry of dental implants has not yet been explicitly investigated in the literature, undersized preparation of the implant osteotomy is recommended at the implants’ tip, while crestally normal drilling protocols are used [45]. This could be of particular relevance for immediate implant placement, since the implant is only fixed in the bone with its apical portion. The present ex vivo study could provide evidence that apically located screw threads compared to an apically rounded implant tip result in an increase in primary stability. It is known that not only the depth but also the shape of the screw threads can influence the primary stability of a dental implant and the peri-implant bone resorption. Thus, it has been shown that occlusal loads after implant placement are mainly concentrated in the bone section adjacent to the first screw winding, indicating that the implant width and the configuration of the windings could lead to a reduction of the loads [14,15,20]. For example, a more square shape of the threads allowed an increase of the bone–implant contact as well as a distribution of the loads on a larger bone surface with a better distribution, especially of lateral forces. A V-shape of the threads in the most apical part of the implant determines a greater aggressiveness of the implant, especially in bone of lower density, achieving a greater mechanical stability of the implant and a greater resistance to vertical forces [14,20,46]. Not only do the observed effects of apically located screw threads on the primary stability of the implant correspond to the current literature, but also fit the mathematical calculations for improving the pressure distribution around the implant by increasing the surface area [14,16,47].

Of course, with regard to the data collected, it must be kept in mind that these are ex vivo data and, therefore, no long-term data on osseointegration, implant survival and success could be obtained. Nevertheless, the study design and the bone quality analyses performed in advance made it possible to study a group that is as uniform as possible and to eliminate confounders that inevitably occur in the living organism. The rather small study population must also be viewed critically; however, it should be noted that the values within the groups remained quite homogeneous. Even though this study made full use of the possibilities available today for analyzing primary stability and attempted to avoid systematic errors by combining the different measurement methods, it cannot be ruled out that the method itself may falsify the results. The different methods have different advantages and disadvantages. The insertion torque, for example, can only be recorded at the time of implant insertion and thus stability monitoring over time is not possible. In contrast, RFA can also be applied for long-term monitoring of dental implant stability [7,10]. However, measurement of insertion torque is believed to be more sensitive for determining primary stability than both RFA and Periotest^®^ values [16,31,48]. Hakim et al. could show that both insertion torque and Periotest^®^ values correlate with bone mineral density measurements by CT, serving as prognostic factor for dental implant primary stability as well as for the implant’s long-term prognosis [49]. Ultrasound characterization as well as resonance frequency tests are influenced by material viscoelasticity and could depend on the frequencies utilized for the tests. For this reason, only one implant system of the same material with the described modifications in the apical screw thread area was used in this study, as well as bone samples that were as homogeneous as possible.

Analogous to other studies in the literature, it could be shown that the use of an undersized drilling protocol results in a significant increase in primary stability [50,51,52], whereby for the first time the additional positive effect of apically located screw threads on primary stability could be demonstrated, especially in soft bone. Overall, caution should be exercised when using undersized drill protocols, as high insertion torques lead to an increased initial implant stability and prevent adverse micromovements under load, but the overcompression of the bone can also jeopardize the healing process via obstruction of angiogenesis, local hypoxia and consecutive necrosis [53]. Consequently, the use of a combination of undersized drilling protocols and changes in (apical) implant geometry—as investigated in the present study—could yield beneficial results, especially in cases of softer bone.

## 5. Conclusions

Altogether, analogous to the existing literature, a strong correlation was shown between implant geometry (for the first time in the literature, also, apically located screw threads), surgical technique, and primary stability. Thus, with increasing implant length in cortico-cancellous bone, an increase in primary stability can be expected, with apically located screw threads on the implant further increasing it. In soft cancellous bone in particular, the apically located screw threads have a further positive influence on primary stability. Thus, it should be noted that modifying factors such as bone density should be taken into account when preparing the implant site and subsequently selecting the implant system.

## Data Availability

Data are available in Tables S1 and S2.

## Supplementary Materials

The following are available online at https://www.mdpi.com/article/10.3390/ma14071728/s1, Table S1: Measurements from Section A and Table S2: Measurements from Section B.
