# Supplementary material for: Does the Modification of the Apical Geometry of a Dental Implant Affect Its Primary Stability? A Comparative Ex Vivo Study
Source: Materials (Basel). 2021 Apr 1;14(7):1728. doi: 10.3390/ma14071728 (PMC8036940; doi:10.3390/ma14071728)
Supplement: Supplementary file 1 [file materials-14-01728-s001.pdf]

materials

Supplementary Materials

Does the Modification of the Apical Geometry of a Dental Implant Affect Its Primary Stability? A Comparative Ex Vivo Study

Henning Staedt [1,2], Diana Heimes [3,*], Karl M. Lehmann [4], Peter Ottl [2,5], Monika Bjelopavlovic [4], Wilfried Wagner [3], Bilal Al-Nawas [3] and Peer W. Kämmerer [3]

[1] Private Practice, Blumenstraße 43, 73728 Esslingen am Neckar, Germany; henning@staedt.com
[2] Department of Prosthodontics and Materials Science, University Medical Center Rostock, Strempelstraße 13, 18057 Rostock, Germany; peter.ottl@med.uni-rostock.de
[3] Department of Oral- and Maxillofacial Surgery, University Medical Center Mainz, Augustusplatz 2, 55131 Mainz, Germany; Wilfried.wagner@unimedizin-mainz.de (W.W.); bilal.al-nawas@unimedizin-mainz.de (B.A.-N.); peer.kaemmerer@unimedizin-mainz.de (P.W.K.)
[4] Department of Prosthetic Dentistry, University Medical Center Mainz, Augustusplatz 2, 55131 Mainz, Germany; karl.lehmann@unimedizin-mainz.de (K.M.L.); monika.bjelopavlovic@unimedizin-mainz.de (M.B.)
[5] Department Life, Light & Matter, University of Rostock, Albert-Einstein-Straße 25, 18059 Rostock, Germany
* Correspondence: diana.heimes@unimedizin-mainz.de; Tel.: +00-49-6131-17-5086

Citation: Staedt, H.; Heimes, D.; Lehmann, K.M.; Ottl, P.; Bjelopavlovic, M.; Wagner, W.; Al-Nawas, B.; Kämmerer, P.W. Does the Modification of the Apical Geometry of a Dental Implant Affect Its Primary Stability? A Comparative Ex Vivo Study. *Materials* **2021**, *14*, 1728. https://doi.org/10.3390/ma14071728

Academic Editor: Yurii Sharkeev

Received: 19 February 2021
Accepted: 29 March 2021
Published: date

Publisher's Note: MDPI stays neutral with regard to jurisdictional claims in published maps and institutional affiliations.

Copyright: © 2021 by the authors. Licensee MDPI, Basel, Switzerland. This article is an open access article distributed under the terms and conditions of the Creative Commons Attribution (CC BY) license (http://creativecommons.org/licenses/by/4.0/).

Table S1. Measurements from Section A.

Measurement Method	Implant Line	Implant Length	Mean (SD)	Confidence Interval
Insertion Torque	J-Line	9 mm	23.25 (0.96)	21.73–24.77
		11 mm	24.75 (1.9)	21.74–27.76
		13 mm	29 (1.82)	26.09–31.91
	K-Line	9 mm	21.5 (1.29)	19.45–23.55
		11 mm	24.5 (0.58)	23.58–25.42
		13 mm	26.75 (0.96)	25.23–28.27
Periotest	J-Line	9 mm	−5.5 (0.58)	−6.42–(−4.58)
		11 mm	−5.5 (0.58)	−6.42–(−4.58)
		13 mm	−4.75 (0.5)	−5.55–(−3.95)
	K-Line	9 mm	−5 (0)	−5
		11 mm	−4 (0)	−4
		13 mm	−4.75 (0.5)	−5.55–(−3.95)
Resonance Frequency Analysis	J-Line	9 mm	68.5 (0.58)	67.58–69.42
		11 mm	77 (0)	77
		13 mm	78.25 (0.5)	77.45–79.05
	K-Line	9 mm	69.5 (1)	67.91–71.09
		11 mm	73.75 (0.5)	72.95–74.55
		13 mm	76.75 (0.5)	75.95–77.55
Push Out Force	J-Line	9 mm	283.75 (14.15)	261.23–306.27
		11 mm	311.75 (5.32)	303.29–320.21
		13 mm	361.25 (9.03)	346.88–375.62
	K-Line	9 mm	260 (8.45)	246.56–273.44
		11 mm	315.5 (7.55)	303.49–327.51
		13 mm	341 (7.57)	328.95–353.05

Table S2. Measurements from Section B.

Measurement Method	Implant Line	Implant Length	Mean (SD)	Confidence Interval
Insertion Torque	J-Line	Normal	17.25 (2.87)	12.68–21.82
		Undersized	37 (5.94)	27.54–46.46
	K-Line	Normal	11.5 (1)	9.91–13.09
		Undersized	26 (0.82)	24.7–27.3
Periotest	J-Line	Normal	−4.75 (0.5)	−5.55–(−3.95)
		Undersized	−6.5 (1)	−8.09–(−4.91)
	K-Line	Normal	−4.42 (0.58)	−4.42–(−2.58)
		Undersized	−4.25 (0.5)	−5.05–(−3.45)
Resonance Frequency Analysis	J-Line	Normal	69 (2.16)	65.56–72.44
		Undersized	72.75 (2.63)	68.56–76.93)
	K-Line	Normal	66.25 (1.5)	63.86–68.64
		Undersized	69.75 (1.71)	67.03–72.47
Push Out Force	J-Line	Normal	285.25 (10.24)	268:95–301.55
		Undersized	365 (8.68)	351.19–378.81
	K-Line	Normal	242.25 (20.82)	209.12–275.38
		Undersized	329 (9.59)	313.74–344.26
